# The Use of Insulin in Surgery
1A Paper read at a meeting of the Bath and Bristol Branch of the British Medical Association on Wednesday, October 27th, 1926.


**Published:** 1926

**Authors:** J. A. Nixon

**Affiliations:** Professor of Medicine in the University of Bristol; Physician to the Bristol Royal Infirmary; Consulting Physician to Southmead Hospital


### THE USE OF INSULIN IN SURGERY.[1]

BY

J. A. Nixon, C.M.G., M.D. Cantab., F.R.C.P.,

*Professor of Medicine in the University of Bristol;*
*Physician to the Bristol Royal Infirmary;*
*Consulting Physician to Southmead Hospital.*

---

Although diabetes and its treatment have been considered to be the province of physicians, surgery has reaped unexpected advantage from the introduction of insulin. The chief causes of death in diabetes are coma and infection. Now coma need not occur in diabetes, that is to say true diabetic coma with acetonæmia and acidosis. There are cases which develop coma from other causes such as uræmia, septicæmia, cerebral hemorrhage, an overdose of narcotics or even meningitis; the last diabetic who died comatose in my wards died of tuberculous meningitis, and we have all seen a cerebral hemorrhage terminate a long-standing case of diabetes.

But true diabetic coma is avoidable. As Joslin says, " The diabetic who dies of coma uncomplicated by infections dies needlessly."

General infections rank high amongst the causes of death. Pneumonia, tuberculosis and influenza certainly head the list, and these cannot as yet be reckoned as coming into the province of surgery.

[1] A Paper read at a meeting of the Bath and Bristol Branch of the British Medical Association on Wednesday, October 27th, 1926.

These general infections, however, cause nearly as high mortality amongst non-diabetics as diabetics.

Local infections appear to be actually of more importance, and perhaps the less severe they appear the less efficient is their treatment. In this connection Joslin remarks, " All are well aware that if a diabetic patient has gall-stones to be removed he instantly commands the services of the leading surgeon on the senior staff, but if a diabetic patient has a sore toe there is no house officer too young to dress it, until a few weeks later, if the patient survives that long, the surgeon in the amphitheatre amputates the thigh."

Minor infections occur frequently amongst diabetics and may affect the course of the disease profoundly. There are many diabetics, especially those who have passed middle life, who by careful dieting alone can live safely. No one wants to embark on an " insulin " regime if it can be avoided. It is not certain that a high blood-sugar is incompatible with good health and long life provided the individual has reached the sixth or seventh decade of life. If, however, such a person should fall a victim to some accidental septic infection the danger is grave. It has been constantly observed that the carbohydrate tolerance is greatly lowered by septic infection. A high blood-sugar renders a person peculiarly susceptible to attack by the ordinary pyogenic organisms. Thus we find diabetics very prone to boils, carbuncles, cellulitis, dental abscess, pyelitis, peri-nephric abscess and the like. It is well recognised that in cases of furunculosis or carbuncles glycosuria must always be looked for, and even if sugar is absent from the urine the amount of sugar in the blood should be investigated. Supposing that a hyperglycæmia is present, should insulin be administered ?

There seems to be a wide-spread impression that once insulin has been administered it will have to be continued throughout life. This is by no means universally true. It happens frequently that although the patient is a potential diabetic with a relatively low carbohydrate tolerance the tolerance is sufficient to allow an adequate diet to be taken without the help of insulin. When infection supervenes a lowered tolerance may result, which has two effects : the first, a lowered tolerance so that an adequate amount of carbohydrate can no longer be metabolised ; and second, that resistance to infection is dangerously decreased. In such a case there must be no hesitation about the use of insulin. The carbohydrate tolerance may rise again after the infection has been successfully resisted and a non-insulin life be resumed. It sometimes happens that a severe degree of diabetes is discovered for the first time during the course of some local infection, such as a carbuncle ; strict dieting in the old-fashioned sense cannot be too vigorously condemned. Deaths from coma most commonly take place when the diet is suddenly changed. If the carbohydrate is restricted and fat and protein simultaneously increased, death from coma may occur within the week. An infection is an additional load for a diabetic to carry ; he needs more, not less, carbohydrate. The only way to enable him to take the extra carbohydrate he requires is to give insulin in proportion to the carbohydrate demand.

There is no need to emphasise the dangers of a high fat diet ; if the carbohydrate tolerance is lowered the dangers of ketosis from an excess of fat over the assimilable carbohydrate are common knowledge.

Gangrene is a complication that calls for separate consideration. It appears in diabetes in two forms.

There is the sudden complete blocking of a large vessel. This is not a diabetic gangrene at all; it is a senile or arterio-sclerotic gangrene occurring in a diabetic patient, who may not survive amputation because of diabetes. Insulin may enable such a patient to stand an operation which in an untreated diabetic would prove fatal, but the cases I have seen and, in some instances, treated with insulin before or after operation have generally died. The other form, which is true diabetic gangrene, results from a local infection to which the soft tissues respond by a rapidly-spreading necrosis. Joslin has pointed out how frequently this form of gangrene is due to injuries occurring to unclean feet. I think it was Joslin who urged that a diabetic should be taught to keep his feet as clean as his face because of this danger. Comparatively slight injuries may lead to severe gangrene. Insulin treatment should never be delayed. Without insulin operation will very likely do no good, with insulin it may prove unnecessary, and if insulin alone does not cure the gangrene it will contribute wonderfully to the success of an operation.

There are certain local infections which may be suspected of being more than mere complications of diabetes; gall-stones, cholecystitis and pericholecystitis have often been suspected to be the exciting focus which has led to a pancreatitis from which diabetes has ensued.

Without expressing the opinion that every such case will benefit from operation, it can be laid down definitely that if other considerations render operation desirable no one should refrain from operating merely because of diabetes.

This brings us to a general consideration of the problem of operations in diabetic subjects. Physicians and surgeons sometimes delay surgical operations in diabetics which in non-diabetics would be performed early and with favourable results. Owing to their poor resistance to infections diabetics as a group require surgery more frequently perhaps than non-diabetics. Where infection is present, as, for example, in appendicitis or pelvic suppuration, diabetics stand in more urgent need of operation than non-diabetics. Infective conditions need even earlier recognition and operation than in non-diabetics, because infection reduces carbohydrate tolerance and predisposes to acidosis.

If time permits of deliberate operation the patient should be given a preparatory course of insulin. I do not confine this advice to major surgical procedures; preparation is no less important in such minor operations as incising boils and carbuncles.

Whenever possible this preparation should be continued for two or three weeks, to allow of restoring the blood sugar to a normal level, to furnishing the patient with an adequate carbohydrate diet and to repairing so far as possible the nutrition.

Here may I make a digression in reference to under-nutrition as a remedial aid in diabetes. In England we have never wholly freed our minds from the superstition of Guelpa that was so ardently adopted by Allen. I refer to the so-called starvation cure of diabetes.

There is still a lingering inclination to bow the knee in the house of famine as well as that of insulin and plenty.

Mosenthal has put the case graphically: "Insulin diminishes the blood-sugar and sets aside glycosuria because it brings about an increased utilisation of glucose, whereas starvation accomplishes this by allowing the accumulated glucose to escape in the urine.

In one case the stranded ship is floated off the sand-bar by the rising tide with its freight intact, while in the other the cargo has to be thrown overboard to enable the vessel to proceed on its way." He agrees, though, that there are exceptional patients in whom the use of a low diet for several days will apparently " activate " the insulin.

But when surgical procedures are contemplated starvation should be avoided like the plague. Insulin brings about that storage of glycogen which is so vitally essential after operation; under-nutrition not only does not add to the store of glycogen, it still further depletes it. No surgeon recognised the dangers of operations in the pre-insulin days more clearly than Treves. " Diabetes," he wrote, " offers a serious bar to any kind of operation . . . a wound in a diabetic patient will probably not heal, while the tissues appear to offer the most favourable soil for the development of putrefactive and pyogenic bacteria. The wound gapes, suppurates and sloughs."

We believe now that it is the high sugar content of the blood that furnishes such a favourable soil to pyogenic organisms. This is almost the only positive harm that can be traced to high blood sugar by itself unless we include cataract. It is little short of miraculous to see sloughing stop, suppuration decrease and the gaping edges of a wound take on a healthy granulation when by means of insulin a high blood sugar is brought back to near normal. No surgeon who has once seen this will want further evidence of the value of insulin in hyperglycæmia.

In another relation to surgery comes the question of anæsthetics. Apart from the gravity of the operation itself diabetics incur great dangers from anæsthesia. Anæsthesia produces a rise in blood-sugar. Some anæsthetics are worse than others in this respect, not one is safe. Chloroform is notoriously the most dangerous and perhaps gas and oxygen the least. Even local anæsthesia has its risks; infiltration anæsthesia may cause extensive necrosis and sloughing. Coma may follow spinal as well as inhalation anæsthesia.

Dr. Flemming was, I think, the first anæsthetist to insist that in a non-diabetic patient pre-operative starvation by itself could produce post-operative acetonæmia and acidosis. What was true of the non-diabetic applies far more forcibly to the diabetic. There is no form of anæsthesia safe for a diabetic until he has been enabled to assimilate an adequate amount of carbohydrate in his diet. It is sugar that protects the diabetic against the dangers of infection, the shock of an operation and post-anæsthetic coma. Insulin, as it were, raises the blockade, and the blood-borne cargo of sugar comes to the relief of the beleaguered tissues.

In post-operative shock there is always present an interference with the internal respiration. Crile believes that the anæsthesia caused by the administration of general anæsthetics is due primarily to this interference. Dr. Fisher, of Boston, Mass., holds that this interference with the internal respiration causes internal acidosis and asphyxia, resulting in lessened oxidation within the cell. By promoting and improving such oxidative processes we can promote the recovery of the body cells and hasten recovery from shock.

When no carbohydrate is available to balance the ketogenic material in the body an excess of ketones is produced. The ketone acids then combine with the alkali of the blood stream and reduce the $CO_2$ combining power. This decrease in the alkali reserve is accompanied by a diminished alkalinity of the blood-stream. These same conditions are present in involuntary fasts, in prolonged vomiting, in cyclic vomiting, after etherisation, in some acute infections, and, of course, in the ketosis of diabetes.

Some interesting observations have recently been made on the blood changes which follow in a Marathon run. The runners who had a normal blood-sugar content at the finish showed no signs or symptoms of shock. The runners who were collapsed at the finish had very low blood-sugars and presented the typical picture of an overdose of insulin. With all this evidence it seems clear that the rational way to combat shock will include giving to the body some substance which will give rise to an immediate supply of energy, and maintaining that supply so long as may be necessary, at the same time furnishing sufficient fluids to keep up the circulating fluid volume. Believing that in the state of shock there is an internal asphyxia and acidosis with oxidative processes held in check, any method for promoting combustion and oxidation and at the same time providing heat-energy should be effective in combating shock.

The diabetic patient enters upon an operation with these oxidative processes already embarrassed; it is small wonder if then diabetes predisposes to post-operative shock.

The combination of glucose with insulin has come to be the established method of treating diabetic and non-diabetic acidosis; the treatment seems to have a settled place in the treatment of post-operative shock in diabetics and non-diabetics.

The first defensive reaction of the organism in shock is the mobilisation of all the available glycogen in the blood-stream to be distributed to the body cells to furnish them with energy; this supply is, however, soon exhausted, hence the replenishing of the glycogen, as by insulin and glucose, thereby becomes of extreme importance in the rational treatment of shock.

In a diabetic untreated before operation the store of glycogen is already fallen dangerously low.

### SUMMARY.

The principal uses of insulin in surgery are :—

1. To enable diabetic patients to combat infection either with or without operation.

2. To render safer for diabetic patients surgical operation whether urgent or deliberate.

3. To protect diabetic patients against the dangers of anæsthesia.

4. To furnish glucose in an oxidisable form to patients, whether diabetic or non-diabetic, who are suffering from shock.

Fisher's method of treating shock is as follows :—

A sterile solution of glucose is used, preferably of 10 to 15 per cent. strength, 500 to 2,000 c.c. may be given depending on the severity of the condition. It should be injected slowly, taking at least an hour, preferably two to four hours (for dilatation of the right heart is a real danger). The amount of insulin injected depends upon the amount of glucose injected. For every 3 grammes of glucose 1 unit of U.20 insulin should be given. The total amount of insulin to be given should be divided into two equal doses, and one part given a quarter of an hour after the intravenous administration has begun, the remainder at the end. So long as there is glycosuria there is no danger of an overdose of insulin. Other drugs may interfere with or influence the effects of this treatment. For instance, adrenalin will counteract the insulin, pituitrin diminishes and sometimes nullifies the sugar-fall, ergotoxin given previously increases the effect of insulin.

### REFERENCES.

[1] E. P. Joslin, *Treatment of Diabetes Mellitus,* Ed. 3, 1924.

[2] H. O. Mosenthal, *Med. Clin. N. Am.,* Philadelphia, 1924-5, viii. 81.

[3] H. O. Petty and W. N. Le Fevre, *Ibid.,* p. 919.

[4] E. P. Joslin, H. F. Root and P. White, *Ibid.,* p. 1,873.

[5] D. Fisher, *Surg. Gynec. and Obst.,* 1926, xliii. 224.

